# Application of Hybrid Fillers for Improving the Through-Plane Heat Transport in Graphite Nanoplatelet-Based Thermal Interface Layers

**DOI:** 10.1038/srep13108

**Published:** 2015-08-17

**Authors:** Xiaojuan Tian, Mikhail E. Itkis, Robert C. Haddon

**Affiliations:** 1 Center for Nanoscale Science and Engineering, University of California - Riverside, Riverside, California 92521, USA; 2 Department of Chemistry, University of California - Riverside, Riverside, California 92521, USA; 3 Department of Chemical and Environmental Engineering, University of California - Riverside, Riverside, California 92521, USA

## Abstract

The in-plane alignment of graphite nanoplatelets (GNPs) in thin thermal interface material (TIM) layers suppresses the though-plane heat transport thus limiting the performance of GNPs in the geometry normally required for thermal management applications. Here we report a disruption of the GNP in-plane alignment by addition of spherical microparticles. The degree of GNP alignment was monitored by measurement of the anisotropy of electrical conductivity which is extremely sensitive to the orientation of high aspect ratio filler particles. Scanning Electron Microscopy images of TIM layer cross-sections confirmed the suppression of the in-plane alignment. The hybrid filler formulations reported herein resulted in a synergistic enhancement of the through-plane thermal conductivity of GNP/Al_2_O_3_ and GNP/Al filled TIM layers confirming that the control of GNP alignment is an important parameter in the development of highly efficient GNP and graphene-based TIMs.

The development of high performance thermal interface materials (TIMs) is an essential part of the progress of the semiconductor industry in increasing the density of electronic circuitry^1^,^2^,^3^. Thermal management technology traditionally utilizes metal or metal oxide fillers of high thermal conductivity to enhance the heat transport across the polymer-based TIM layers which fill the thermal interfaces formed within the hierarchy of the electronic packaging architecture^2^,^3^,^4^,^5^. In the last decade, carbon based nanostructured materials, such as one-dimensional (1D) carbon nanotubes (CNTs) and two-dimensional (2D) graphene and graphite nanoplatelets (GNPs), have attracted a significant interest because of their extremely high intrinsic thermal conductivity along the graphene-based layers thereby facilitating the formation of efficient heat conducting networks within the polymer matrix^1^,^5^,^6^,^7^,^8^,^9^,^10^,^11^,^12^,^13^,^14^,^15^,^16^.

Recently, we observed that the predominantly in-plane alignment of 2D GNPs in thin TIM layers resulted in an increased in-plane electrical and thermal conductivity at the expense of the through-plane transport properties which were significantly suppressed^16^. GNP and boron nitride 2-D filler alignment has been reported by several groups^11^,^13^,^17^,^18^, and has been found to be beneficial for some electronic packaging applications such as heat spreaders^3^. However, in many cases the heat should be transferred across the TIM layers, so the suppression of the through-plane component of the heat conductance is detrimental for the performance of graphene-based fillers in thermal management applications. A promising direction for the enhancement of through-plane thermal conductivity is associated with utilization of vertically grown carbon nanotubes or aligned carbon fibers, but it remains to achieve a sufficient loading of the filler in this configuration^19^,^20^.

The utilization of multicomponent fillers which include particles of different shapes and dimensions is a common practice in the manufacture of commercial TIMs^21^,^22^,^23^. Mostly, this is directed towards a more complete filling of the voids which must be occupied by the thermal interface layer including micro(nano)-cavities formed due to roughness of the contacting surfaces of the electronic modules and the electronic packaging components. In our recent work we combined 1D CNTs and 2D GNPs to formulate a hybrid filler which showed a synergistically enhanced bulk thermal conductivity as a result of decreasing inter-particle junction resistance within the hybrid heat conducting network^10^. In this manuscript we combine GNPs with conventional low aspect ratio aluminum (Al) and aluminum oxide (Al_2_O_3_) micro-particle fillers in order to suppress the in-plane alignment of the GNP filler and enhance the through-plane component of the heat conductance.

## Results

**Figure 1:**
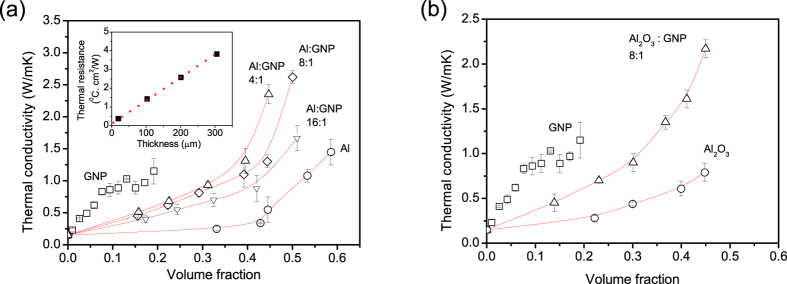
Through-plane thermal conductivity of the thermal interface layers. (**a**) GNP-Al and (**b**) GNP-aluminum oxide hybrid fillers of different formulations as a function of the volume fraction of the hybrid filler. Inset in (**a**) shows an example of the dependence of the thermal resistance of the composite layer on the layer thickness utilized for calculation of through-plane electrical conductivity (composite with 7 Vol% GNP).

In order to improve the through-plane thermal transport across the thermal interface layer 2D-GNPs were combined with conventional low aspect ratio aluminum (Al) and aluminum oxide (Al_2_O_3_) micro-particle fillers. These fillers were chosen for their low cost and good thermal performance in traditional thermal interface materials. The size of isotropic spherical Al and Al_2_O_3_ microparticles was chosen to match the average lateral dimensions of ~10 μm (aspect ratio of  ~ 300) of highly anisotropic GNPs^12^ which intuitively should best serve to disrupt the in-plane alignment of the GNPs. The resulting thermal composites were characterized by a variety of techniques as a function of the hybrid filler formulation. Multiple individual and hybrid fillers formulations were tested and the resulting values of the thermal conductivities are presented in Fig. 1. The maximum practical filler loading was limited by the viscosity and spreadability of the thermal paste. The thermal conductivity was calculated from the slope of the dependence of thermal resistance on grease layer thickness (Fig. 1a, inset). For the single component Al micro-particle filler we observed typical percolation behavior with a strong upturn in the thermal conductivity in the 45–60 Vol% loading range at which point the concentration of filler particles became sufficient to form a percolating thermally conducting network across the polymer matrix, in agreement with literature data^24^.

At low filler loadings (below 10 Vol%), the single component GNP filler is much more efficient than Al and Al_2_O_3_ spherical particles showing a linear increase of thermal conductivity with increasing filler loading in agreement with previous reports^7^,^25^. However, the increasing viscosity and reduced spreadability of the paste limits the GNP loading to ~15–20 Vol% and within this range no sign of percolation is observed. The absence of percolation can be attributed to the high interparticle junction resistance in comparison with the intra-particle thermal resistance^26^.

Hybrid filler formulations with volume proportions of Al and GNP fillers of 4:1, 8:1 and 16:1 produce greases which combine the advantages of the individual fillers as evidenced by the increasing slope of the dependence of thermal conductivity on the filler loading in the low loading range and the percolation-induced sharp increase of thermal conductivity at high loadings (Fig. 1a). The percolation threshold for hybrid fillers is lower than that of individual Al or Al_2_O_3_ fillers because of the high aspect ratio of GNP component. The highest gain in performance was achieved for an 8:1 Al to GNP volume ratio formulation resulting in a through-plane thermal conductivity of the grease, κ_┴_ = 2.6 W/mK, which is almost two times higher than the composites with individual fillers of similar viscosity and spreadability. Substitution of aluminum with aluminum oxide filler provide qualitatively similar results (Fig. 1b), but with less pronounced percolation behavior, more likely, due to the almost order of magnitude lower intrinsic thermal conductivity of aluminum oxide. Nevertheless, aluminum oxide may have some other advantages because it can provide an electrically insulating thermal grease^15^,^27^,^28^.

Below, we examine the origin of the improved performance of the hybrid fillers and evaluate the correlation of the observed enhancement of thermal conductivity with the degree of GNP in-plane alignment and its suppression upon addition of the spherically shaped filler.

**Figure 2:**
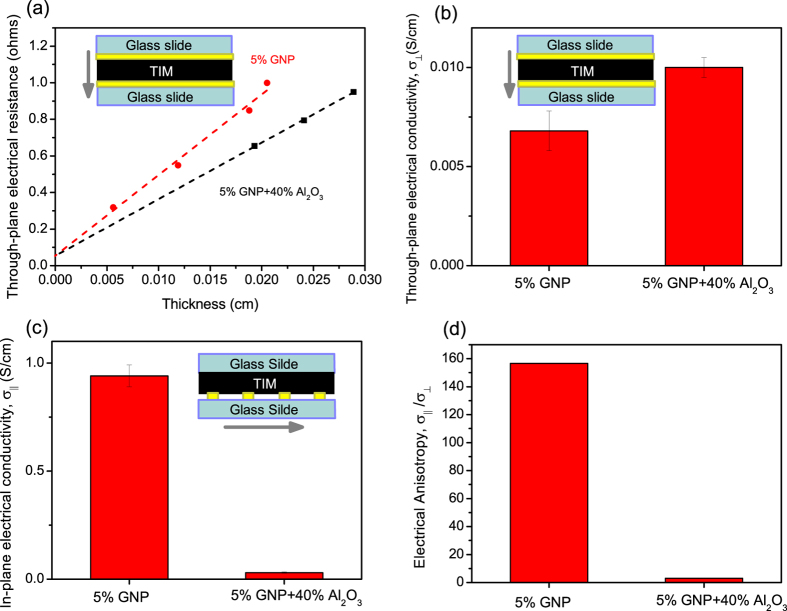
Measurements of anisotropic electrical properties of GNP and GNP/Al_2_O_3_ hybrid composites. (**a**) Dependence of the 2 probe through-plane electrical resistance of TIM layer on layer thickness. (**b**) Through-plane, and (**c**) in-plane, electrical conductivities of GNP and GNP/Al_2_O_3_ hybrid composites. Insets show schematics of the electrical measurements. (**d**) Electrical anisotropy of the composites.

Our recent study demonstrated that the natural in-plane alignment of GNPs during formation of TIM layer leads to a high electrical anisotropy of the electrical conductivity with a ratio of in-plane (σ_‖_) to through-plane (σ_┴_) components exceeding 1000^16^. Thus we used electrical anisotropy to qualitatively follow the disruption of the in-plane alignment of GNPs upon addition of spherically shaped filler particles. Electrically insulating Al_2_O_3_ particles of diameter 1–10 μm were utilized for the electrical anisotropy measurements so that only the contribution of the electrically conductive GNP filler is assessed. Through-plane and in-plane electrical conductivity measurements were conducted on the layers of thermal grease placed on the sample stage of the TIM tester between two glass slides under the same pressure and temperature as in case of the thermal conductivity measurement so as to closely reproduce the formation of a TIM layer under normal operating conditions. The schematics of the 2-probe through-plane and 4-probe in-plane electrical conductivity measurements are presented in Fig. 2a (inset) and Fig. 2c (inset), respectively. Figure 2b,c show the through-plane and in-plane electrical conductivities of the greases with two filler formulations: i) thermal grease with a single filler of 5 Vol% GNP loading, and ii) thermal grease containing a hybrid filler combining 5 Vol% GNP and 40 Vol% Al_2_O_3_, and Fig. 2d shows the electrical anisotropy of these greases. Greases with individual GNP (5 Vol%) fillers is strongly anisotropic with σ_‖ _= 0.94 S/cm and σ┴ = 0.006 S/cm, and a corresponding anisotropy ratio σ_‖_/σ_┴_ ~ 160.

The hybrid filler grease shows increased through-plane conductivity σ_┴_ = 0.01 S/cm (Fig. 2b) and significantly decreased in-plane conductivity σ_‖ _= 0.03 S/cm, which corresponds to a strongly decreased anisotropy, σ_‖_/σ_┴_ ~ 3. It should be noted that addition of alumina particles could affect the degree of GNP dispersion, rheology and the microstructure of the composite grease and, ultimately, the formation of percolating networks defining the electrical and thermal transport. However, the changes in GNP dispersion and composite microstructure, are likely to be isotropic in nature and should affect in-plane and through-plane components of electrical conductivity in a similar way while the experimentally observed changes are of opposite signs corresponding to increasing through-plane and decreasing in-plane components (Fig. 2). This suggests that the modification of the composite anisotropy probably involves a change of orientation of GNPs with the enhancement of the through-plane component and we provide further information on these orientation changes in the SEM study (below).

**Figure 3:**
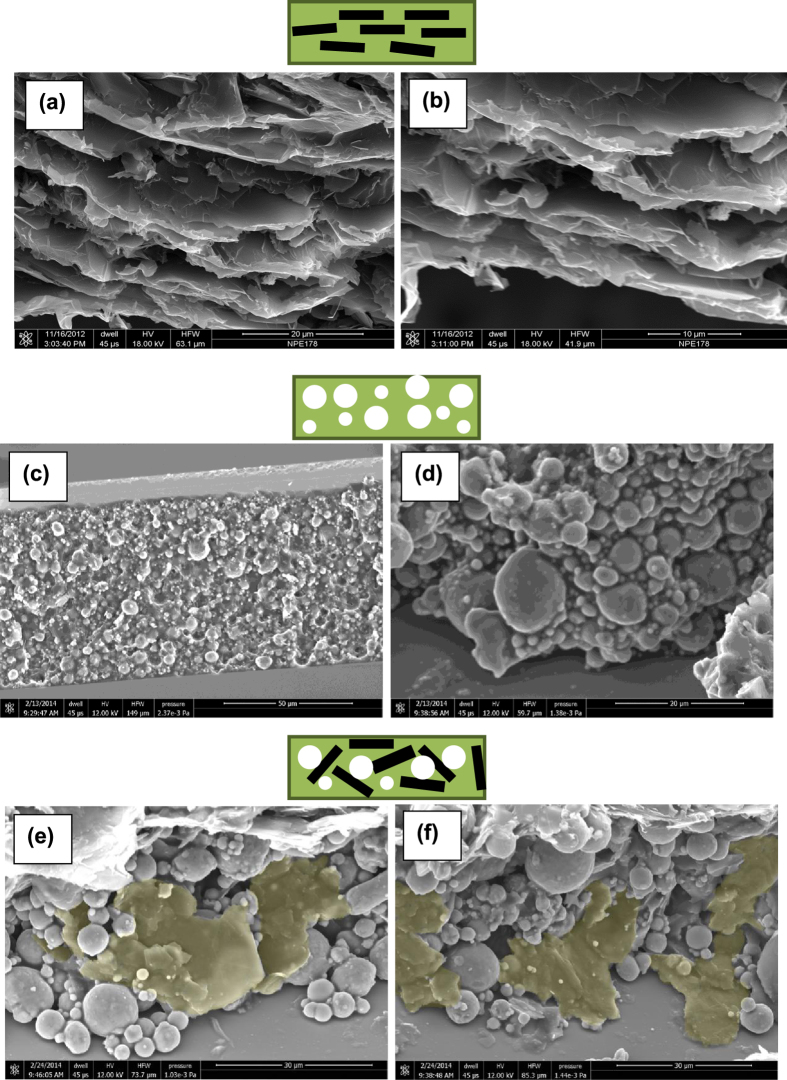
SEM imaging of cross-sectional fracture surfaces of composites with different fillers. (**a**) and (**b**) GNP filler; (**c**) and (**d**) alumina filler; (**e**) and (**f**) hybrid GNP/Al_2_O_3_ filler (45 Vol% of hybrid filler with the ratio of Al_2_O_3_ to GNP as 8:1). Schematics of filler particles orientation are presented on top of the SEM images. Scale bars: (**a**) 20 μm; (**b**) 10 μm; (**c**) 50 μm; (**d**) 20 μm; (**e**) 30 μm; (**f**) 30 μm.

Scanning electron microscopy (SEM) imaging allows the direct observation of the orientation of GNPs in the TIM layer. Cross-sectional fracture surfaces for SEM study were prepared by curing the original grease samples under physical conditions which closely reproduce the parameters of formation of the thermal interface layer, followed by freezing the cured samples and fracturing with shear force (see methods section). The cross-sectional SEM images of a range of different composites are presented in Fig. 3.

Figure 3a,b reveal that majority of GNPs form a structure aligned in the plane of the thermal interface layer in agreement with our previous report^16^. Figure 3c,d show the Al_2_O_3_ filler which adopts a uniform, isotropic distribution of spherical particles of sizes 1–10 μm. Figure 3e,f represent a cross-sectional area of the composite with the hybrid filler. It can be noticed that the microstructure of the fracture surface changes in comparison with the case of single GNP filler (Fig. 3a,b): a tendency for separation of individual GNPs in the presence of the spherical filler can be observed (Fig. 3e,f) with the a number of the GNPs showing a significant out-of-plane orientation in agreement with the observed decrease in anisotropy of the electrical conductivity. In principle, the addition of the curing agent can modify the microstructure of the composite but it is unlikely that it would dramatically change the orientation of GNPs under carefully controlled preparation conditions.

## Discussion

**Figure 4:**
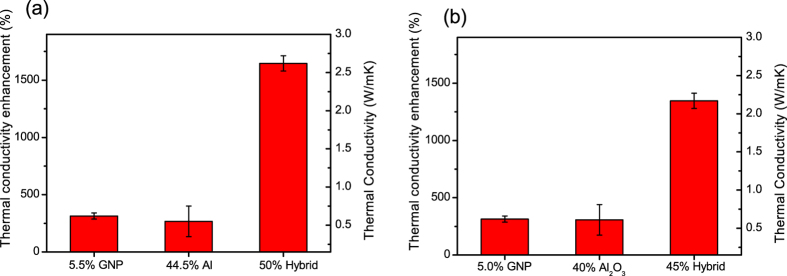
Through-plane thermal conductivity enhancement and thermal conductivities of hybrid fillers composites. (**a**) GNP/Al formulations; (**b**) GNP/Al_2_O_3_ formulations.

Thus the conducted study of anisotropic electrical and heat transport properties and microscopic analysis of the cross-sectional morphology of the hybrid TIM layers clearly indicate that addition of spherical microparticles leads to the disruption of the GNP in-plane alignment. Figure 4 summarizes the effect of hybrid filler formulation on the improvement of the through-plane thermal conductivity of the thermal greases in terms of thermal conductivity and thermal conductivity enhancement, TCE = (κ_┴_ – κ_0_)/κ_0_, %, where κ_0_ is thermal conductivity of uncured epoxy matrix, 0.15 W/mK, and κ_┴_ corresponds to the thermal conductivity of the particular filler formulation.

As shown in Fig. 4a, a GNP filler of loading 5.5 Vol% provides a TCE of ~300% (κ_┴_ = 0.62 W/mK), an Al filler of loading 44.5 Vol% provides TCE of ~270% (κ_┴_ = 0.55 W/mK), whereas combined, these loadings provide TCE of ~1650% (κ_┴_ = 2.62 W/mK) which is more than twice the simple sum of the individual fillers contributions, thus showing the synergistic character of the enhancement. Similar results were obtained for combination of GNP and Al_2_O_3_ filler as shown in Fig. 4b. This synergistic enhancement of the through plane thermal conductivity of the TIM layer is in correlation with the disruption of GNPs in-plane alignment following the addition of the spherical filler, as confirmed by electrical anisotropy and SEM studies, leading to the improved through-plane percolating pathways for the heat transport. Performance of the hybrid filler can be further improved by tuning the filler formulation and decreasing the interparticle thermal resistance along the filler network and at the filler-polymer matrix interfaces, for example, by chemically modifying the surface of the filler components. This would allow achieving high TIM performance at lower filler loading in the range of optimum viscosity and spreadability of the thermal grease^29^.

In summary, disruption of the in-plane alignment of GNPs in thin thermal interface layer was achieved by addition of conventional spherical shape Al_2_O_3_ or Al filler particles. This disruption was confirmed by studies of the electrical anisotropy and SEM imaging. This hybrid filler formulation resulted in a synergistic enhancement of the through-plane thermal conductivity of the hybrid GNP/Al or GNP/Al_2_O_3_ based TIM layer. The enhancement of the out-of-plane orientation of GNPs therefore emerges as an important tool for expanding the potential of GNPs and other 2D fillers to contribute to high performance thermal management applications.

## Methods

### Preparation of GNPs based thermal grease

In a typical experiment, 30 ml of concentrated nitric acid was carefully added to 90 ml of concentrated sulfuric acid and the mixture was stirred for 10 min. Then natural graphite (10 g, TIMCAL, 300 μm particle size) was added into the acid mixture, stirred for 10 mins at 600 rpm and left overnight to allow acid intercalation of the graphite. The acid was removed by vacuum filtration of the mixture utilizing Millipore glass filter holders for 47 mm disc filters with PTFE membrane (Millipore, 0.2 μm FG) and house vacuum; the excess acid was washed from the membrane with DI water until the pH of the filtrate was neutral. The collected graphite was dried in air at room temperature for 2–3 days. For thermal shock exfoliation 2 g of the intercalated graphite was loaded in a quartz boat, inserted into the cold zone of a quartz tube mounted in a Linberg Blue tube furnace under argon flow and the boat was shifted rapidly into the 800 ^o^C hot zone and soaked for 3 mins to produce expanded graphite. GNPs were obtained by dispersing expanded graphite in acetone (200 mg in 100 ml) by shear mixing (Fisher Scientific, Power GEN 500, 42000 RPM) for 30 min followed by sonication (bath sonicator, VWR Scientific model 550HT) for 24 hr. Epoxy 813 (Epon^TM^) was added into the GNPs dispersion followed by shear mixing (Fisher Scientific, Power GEN 500, 42000 RPM) for 30 mins. Acetone was removed by overnight aeration. The resulting grease was homogenized in a dual asymmetric centrifuge Speed Mixer (DAC 150.1 FVZ, Flack Tek, Inc.) at 3200 rpm for 3 min. Efficient mixing is achieved as a result of the double rotation of the mixing cup which produces strong centrifugal shear forces. For hybrid filler based thermal greases, aluminum (4.5 ~ 7 μm, Alfa Aesar) or aluminum oxide (1–10 μm, Sigma-Aldrich) spherical shape fillers were added into the GNP-based grease and homogenized by the speed mixer with a variety of different proportions of the individual fillers to give a filler loading in the range of 0–60 Vol%. The densities used in the calculations are as follows: GNPs (2.26 g/cm^3^), Al_2_O_3_ (4 g/cm^3^), Al (2.7 g/cm^3^), epoxy matrix 813 (1.14 g/cm^3^).

### Thermal conductivity measurement

The through-plane thermal conductivity of the GNP based greases was measured with a LW-9389 TIM Tester (Longwin, Taiwan) utilizing the steady-state heat flow technique according to ASTM D5470-06^16^. The heat flow and the temperature across the TIM layer was measured utilizing a set of precision thermometers positioned in copper metal blocks along the direction of the thermal gradient. Thermal resistances of thin layers of lateral dimensions of 2.5 cm × 2.5 cm of 3 or 4 different thicknesses varying from 20 μm to 400 μm were measured and the bulk through-plane thermal conductivity was calculated from the slope of the thermal resistance vs thickness dependence thus allowing the exclusion of the contact thermal resistances at the interfaces between the thermal grease and the copper blocks.

### Electrical conductivity measurements

In-plane and through-plane electrical conductivities of the thermal grease were measured following the recently reported procedure^16^. For the in-plane electrical conductivity measurements the thermal grease was spread between two glass slides with spacer controlling 75 μm thickness of the layer. 4 in-line gold electrodes were patterned 5 mm apart on the bottom slide and the 4-probe resistance was measured using a 2400 Keithley Measure Unit. A 2-probe technique was applied for through-plane electrical conductivity measurements: the electrical resistances of grease layers of three different thicknesses in the range between 50 and 300 μm was measured by sandwiching the grease layer between two gold coated glass slides of contact area 2.5 cm × 1.25 cm to allow the calculation of the bulk through-plane electrical conductivity from the slope of a resistance vs thickness plot, thereby excluding the contact resistance. For both in-plane and through-plane electrical conductivity measurements the applied pressure, 40 psi, and the temperature of the grease, 60 ^°^C, match the conditions of typical TIM applications and thermal conductivity measurements. The advantage of this technique is that it is applied *in situ* to the grease layer positioned between the heat flow transmitting blocks of the TIM Tester, so the grease layer is formed under the same pressure and temperature as in the grease composite thermal conductivity testing.

### Scanning Electron Microscopy (SEM) study

The SEM study was conducted utilizing Nova NanoSEM 450 instrument (FEI Company). To obtain a well-defined cross-sectional image of the filler particles, the epoxy based samples containing individual GNP and Al_2_O_3_ spherical fillers and also the hybrid GNP/Al_2_O_3_ materials were cured after addition of the curing agent (EPI-KURE W from EPON^TM^) to the grease formulation. The materials were carefully cured in order to preserve as much as possible the conditions of the TIM layer formation and testing. In particular, for the thermal conductivity measurements we utilized a commercial TIM tester (Longwin LW-9389 TIM Tester, Taiwan) in which the composite grease layer is placed under controlled pressure 40 psi between two copper blocks generating a small temperature gradient (ΔT ~ 1–3 ^°^C) at average temperature of 60 ^°^C. Thus the TIM Tester itself represents a small hot press of controlled pressure and temperature. For curing we placed the composite sample in the hot press under the same pressure and temperature as the grease sample thus reproducing the condition of the grease layer formation and testing as closely as possible. Cross-sections of the resulting cured thin films were prepared by immersing the films in liquid nitrogen and fracturing the frozen samples with shear force.

## Additional Information

**How to cite this article**: Tian, X. *et al*. Application of Hybrid Fillers for Improving the Through-Plane Heat Transport in Graphite Nanoplatelet-Based Thermal Interface Layers. *Sci. Rep*. **5**, 13108; doi: 10.1038/srep13108 (2015).
